# Supplementary material for: Obstructive Sleep Apnoea Severity Is Negatively Associated with Depressive Symptoms: A Cross-Sectional Survey of Outpatients with Suspected Obstructive Sleep Apnoea in Japan
Source: Int J Environ Res Public Health. 2022 Apr 20;19(9):5007. doi: 10.3390/ijerph19095007 (PMC9099749; doi:10.3390/ijerph19095007)
Supplement: Supplementary file 1 [file ijerph-19-05007-s001.zip › ijerph-1659786-supplementary.pdf]

Table S1. Logistic regression analysis: comparison between patients with AHI/REI <15 and ≥ 15.

	Unadjusted odds ratio (95% CI)	*P*	Adjusted odds ratio (95% CI)	*P*
Age (years)	1.04 (1.03–1.04)	<0.001	1.04 (1.03–1.05)	<0.001
Male (%)	2.27 (1.72 – 3.00)	<0.001	2.35 (1.70–3.24)	<0.001
BMI (kg/m^2)	1.11 (1.08–1.14)	<0.001	1.14 (1.10–1.18)	<0.001
Snoring (%)	1.10 (0.86–1.42)	0.452	1.11 (0.83–1.48)	0.488
ESS	0.95 (0.93–0.98)	<0.001	1.02 (0.99–1.05)	0.221
AIS	0.94 (0.91–0.97)	<0.001	1.00 (0.96–1.04)	0.937
PHQ-9	0.93 (0.90–0.95)	<0.001	0.96 (0.92 – 1.00)	0.042
Hypertension (%)	3.01 (2.33–3.90)	<0.001	1.47 (1.07–2.03)	0.017
Diabetes (%)	1.79 (1.30–2.46)	<0.001	0.89 (0.61–1.22)	0.547
Hyperlipidaemia (%)	1.53 (1.15–2.02)	0.003	0.88 (0.64–1.22)	0.451
Cardiovascular diseases (%)	1.69 (1.11–2.58)	0.014	0.85 (0.53–1.37)	0.504
Cerebrovascular diseases (%)	2.16 (1.19–3.92)	0.012	1.36 (0.71–2.60)	0.357
Mental disorders (%)	0.46 (0.33–0.64)	<0.001	0.81 (0.53–1.26)	0.355

BMI, body mass index; PHQ-9, Patient Health Questionnaire-9; AIS, Athens insomnia scale; ESS, Epworth sleepiness scale; AHI, apnoea-hypopnea index; REI, respiratory event index.
